# Computational Modeling of Single Neuron Extracellular Electric Potentials and Network Local Field Potentials using LFPsim

**DOI:** 10.3389/fncom.2016.00065

**Published:** 2016-06-28

**Authors:** Harilal Parasuram, Bipin Nair, Egidio D'Angelo, Michael Hines, Giovanni Naldi, Shyam Diwakar

**Affiliations:** ^1^Amrita School of Biotechnology, Amrita Vishwa Vidyapeetham (Amrita University)Amritapuri, India; ^2^Department of Brain and Behavioral Sciences, University of PaviaPavia, Italy; ^3^Brain Connectivity Center, C. Mondino National Neurological InstitutePavia, Italy; ^4^Department of Neuroscience, Yale School of MedicineNew Haven, CT, USA; ^5^Department of Mathematics, University of MilanMilan, Italy

**Keywords:** Local Field Potential, simulation, neuron, circuit, computational neuroscience, cerebellum, neocortex

## Abstract

Local Field Potentials (LFPs) are population signals generated by complex spatiotemporal interaction of current sources and dipoles. Mathematical computations of LFPs allow the study of circuit functions and dysfunctions via simulations. This paper introduces LFPsim, a NEURON-based tool for computing population LFP activity and single neuron extracellular potentials. LFPsim was developed to be used on existing cable compartmental neuron and network models. Point source, line source, and RC based filter approximations can be used to compute extracellular activity. As a demonstration of efficient implementation, we showcase LFPs from mathematical models of electrotonically compact cerebellum granule neurons and morphologically complex neurons of the neocortical column. LFPsim reproduced neocortical LFP at 8, 32, and 56 Hz via current injection, *in vitro* post-synaptic N_2a_, N_2b_ waves and *in vivo* T-C waves in cerebellum granular layer. LFPsim also includes a simulation of multi-electrode array of LFPs in network populations to aid computational inference between biophysical activity in neural networks and corresponding multi-unit activity resulting in extracellular and evoked LFP signals.

## Introduction

Extracellular recording is a classical method used to study neuronal behavior at the population level (Caton, 1874). Studies based on this technique have contributed significantly to the current understanding of network behavior (Mitzdorf, 1985; Di et al., 1990; Kandel and Buzsáki, 1997; Scherberger et al., 2005; Montgomery and Buzsáki, 2007; Colgin et al., 2009). Biophysical computations underlying extracellular recording technique have aided the development of reliable quantitative mathematical models that elucidate the generation of extracellular potential (LFP) from transmembrane ionic currents (Gold et al., 2007). The forward modeling schema was developed in 1960s and used transmembrane ionic currents to calculate extracellular field potential from biophysical neuronal models (Rall and Shepherd, 1968; Plonsey, 1969). The technique has been validated using single neuron models (Holt and Koch, 1999; Gold et al., 2006; Diwakar et al., 2011) and network models (Lindén et al., 2011; Reimann et al., 2013).

An extracellular microelectrode or “LFP electrode” is comprised of a sharp metal or solution-filled glass tip at one end with the line end connected to a data acquisition system. The extracellular electrode has been known to record voltage fluctuations from the extracellular medium generated by spatially inhomogeneous transmembrane currents related to neuronal processes (Eccles, 1951). The relative range of a microelectrode is reported in the range of 0–10 kHz and attributed to sources (neuronal processes) within the vicinity of the electrode (Egert et al., 2002; Buzsaki, 2006; Lindén et al., 2011). In the case of evoked post-synaptic activity, Local Field Potential (LFP) is the signal recorded below 500 Hz (low-frequency component) and high frequency component (>500 Hz) has been referred as Multi Unit Activity (MUA) (Buzsáki, 2004). LFPs are population signals presumed to be composed of several components, including synaptic transmembrane current, action potentials and sodium currents, calcium spikes, and ephaptic contacts (Buzsáki et al., 2012; Einevoll et al., 2013; Mineault et al., 2013).

Popular models for extracellular current sources include the Point Source Approximation (PSA; Rall and Shepherd, 1968; Holt and Koch, 1999), Line Source Approximation (LSA; Gold et al., 2006), and low-pass RC filter (Bédard et al., 2004). These have been used along with the forward modeling schema to calculate the extracellular field potential generated by neuronal process at distance “r.” These techniques vary in their theory of estimation of field potentials from transmembrane synaptic currents. LSA generally displays more accuracy in the prediction of field potential except at a close distance, less than a micron away from the sources (Rosenfalck, 1969; Trayanova et al., 1990; Gold et al., 2006). Detailed biophysical understanding of the generation of this ensemble response has implicated this signal (LFP) as a valuable technique to study network function. LFP recordings have also been known to help connecting hand movement patterns to underlying neuronal mechanisms with implications on the development of neuroprosthetic devices (Mehring et al., 2003; Rickert et al., 2005).

We developed a script-based GUI tool, LFPsim in order to facilitate computation of extracellular potential in simulations of conductance based cable models of neurons and networks (Migliore et al., 2003; Hines et al., 2004; Gleeson et al., 2012). LFPsim is a plug-in script developed toward this goal of modeling and analysis of extracellular activity using NEURON simulation environment (Hines and Carnevale, 1997). Tools like ViSAPy (Hagen et al., 2015), LFPy (Lindén et al., 2014), VERTEX (Tomsett et al., 2015) require reimplementation of some models. Our goal was to allow reuse of NEURON (Hines and Carnevale, 2000) models and to allow constraining multicompartmental models via LFPs (Gold et al., 2007). LFPsim was developed to compute electric potential of single neuron, population LFP and their spatio-temporal dynamics using existing and new models in NEURON and available on ModelDB.

## Methods

### Overview of LFPsim

LFPsim was designed as an easy-to-use tool for modeling and computing extracellular electric field of single neuron and LFP of a population of neurons. LFPsim uses NEURON's extracellular mechanism to calculate total ionic currents from neuronal compartments. Three biophysical modeling schemas were implemented to model the extracellular activity. The field potential was calculated using PSA, LSA (Holt and Koch, 1999; Gold et al., 2006) and Resistance-Capacitance (RC) based low-pass filter techniques (Bédard et al., 2004). Extracellular potential at each time step (dt) was calculated by setting pointers to “lfp.mod” and for multiple recording points, “mea.mod.” “lfp.mod” and “mea.mod” are NMODL files used by the LFPsim to calculate extracellular potential generated by individual current sources at each time step.

### Extracellular potential and cable compartments

Extracellular field potentials were computed from transmembrane ionic currents in the extracellular medium (Reimann et al., 2013). Transmembrane ionic currents are summed active currents generated from individual ion channels of neuronal processes and they diffuse into extracellular medium when action potential propagate in the neuron.

**Figure 1:**
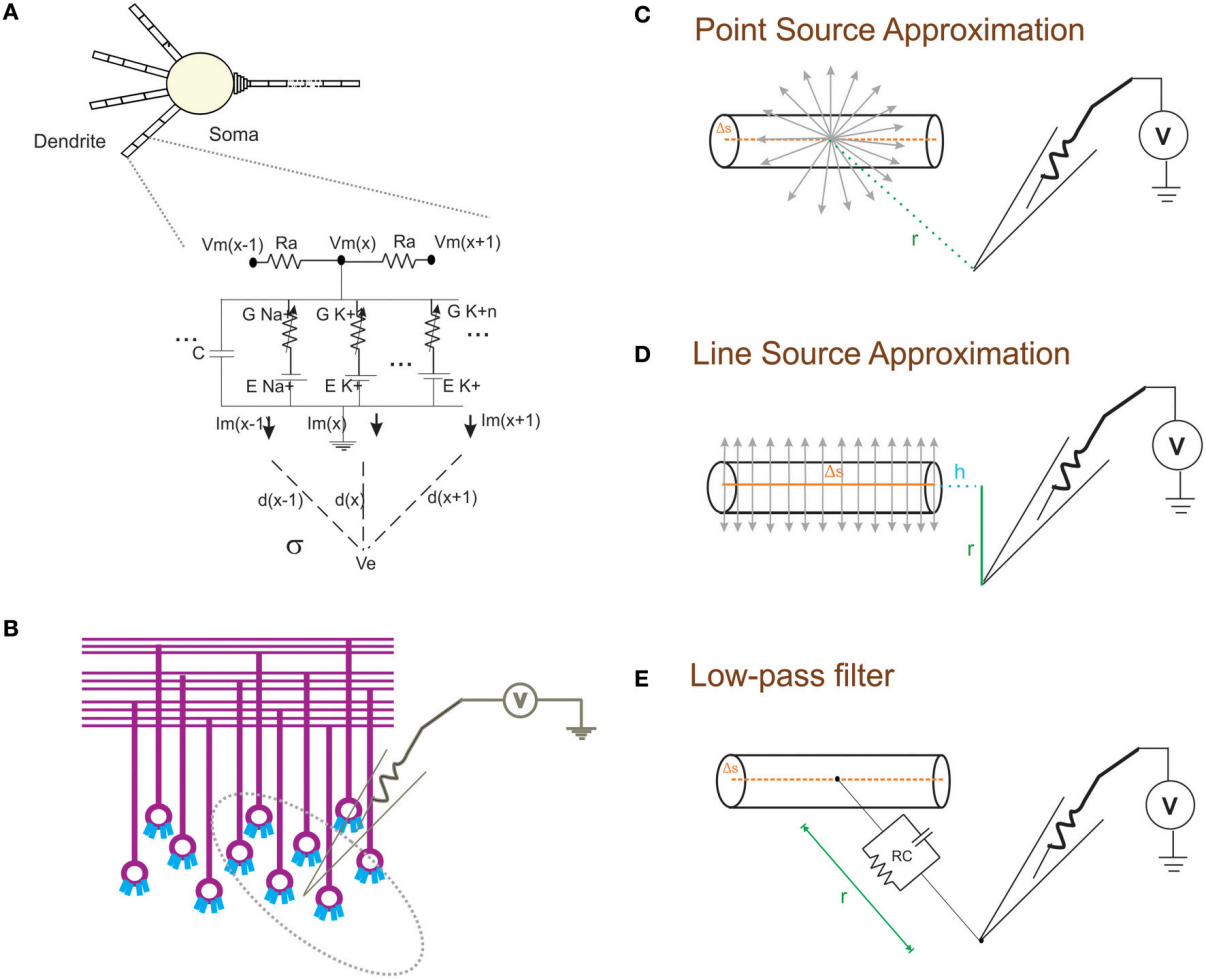
**Schematic of modeling extracellular potential and recording from single neuronal compartment and neuronal ensembles**. **(A)** Electrical equivalent circuit of granule cell model (Diwakar et al., 2009) with extracellular mechanism; Two layers of RC compartments added at the dendrite (indicative representation). **(B)** Cartoon of LFP electrode (microelectrode) recording from cerebellar granular layer ensemble. Schematic representation of different approximation techniques **(C–E)** used to model extracellular potential from detailed biophysical model of a neuronal compartment. **(C)** Each electrical source related to the neuronal compartment in Point Source Approximation (PSA) technique, is assumed to be at the center of the neuritic process. **(D)** In the Line Source Approximation (LSA) method, the electrical source was modeled as a line passing through the center of the neuritic volume. **(E)** The extracellular medium's low pass filtering properties was modeled as a Resistance-Capacitance (RC) filter.

With NEURON, the transmembrane ionic currents in multi-compartmental models can be calculated by summing up all active currents estimated using extracellular mechanism from NEURON as implemented in LFPsim (see Equation 1). Circuit representation of intracellular and extracellular potentials is shown in Figure 1A. Transmembrane ionic currents are approximated such that the total current through a compartment is equal to the current density as the center of the compartment times the membrane area of the compartment (Segev et al., 1989; De Schutter, 2010).

(1) $$\begin{array}{l} I_{transmemberane}=I_{ionic}+c_{m}\frac{\partial V_{m}}{\partial t} \end{array}$$

Where, *I*_*ionic*_ represent the ionic currents and $c_{m}\frac{\partial V_{m}}{\partial t}$ represent the capacitive current.

The forward modeling schemas (Rall and Shepherd, 1968; Holt and Koch, 1999) allow modeling the extracellular field potential around neurons (Figure 1B). The computational schemas of the LFPsim involve two steps. (1) Estimation of total ionic current from detailed biophysical model of neuronal compartments. (2) Calculation of field potential at a point, P(x,y,z) from calculated ionic currents. In LFPsim, computations were estimated for each time step.

### Modeling extracellular potential from transmembrane current

From individual current sources, the extracellular potential was computed using PSA, LSA (Holt and Koch, 1999; Gold et al., 2006) and RC low pass filter techniques, implemented within LFPsim.

Studies (Rall and Shepherd, 1968; Bédard et al., 2004; Gold et al., 2006) indicate neuropil can be modeled as an isotropic volume conductor with no capacitive effect of the medium for frequency range (0–3000 Hz). This allows the extracellular medium to be described as a purely ohmic conductivity (Plonsey, 1969; Holt, 1998).

In quasistatic approximation of Maxwell's equation, the electric field (E) and magnetic field (B) are effectively decoupled (Hämäläinen et al., 1993). ∇ ^*^E = 0, the electric field in the extracellular medium can be related to extracellular potential (Φ) by E = −∇Φ. A linear relationship was assumed between the transmembrane current density (J_m_) and the extracellular field potential (see Equation 2, Hämäläinen et al., 1993). σ denotes the extracellular conductivity.

(2) $$\begin{array}{l} \sigma \nabla \Phi ={\text{J}}_{\text{m}} \end{array}$$

Extracellular potential generated from a neuronal compartment or segment can be generally approximated as either to a point or line source (Holt and Koch, 1999; Gold et al., 2006). Point source models assume transmembrane currents are generated from a point at the center of the neurite (see Figure 1C). Currents from each compartment across three dimensional space were computed from individual point sources. In the first schema, PSA technique was implemented in LFPsim to estimate the extracellular waveform. For a single point source, “*I*” denotes the transmembrane current generated from the source, “σ” denotes the conductivity of the medium and “*r*” denotes the distance from source to the point of measurement. Extracellular potential (Φ) at a distance “*r*” is computed as

(3) $$\begin{array}{l} \Phi =\frac{I}{4\pi \sigma r} \end{array}$$

Considering “*n*” point sources in the extracellular medium, LFP was computed as,

(4) $$\begin{array}{l} \Phi_{LFP}=\overset{n\text{\_}sources}{\underset{i=1}{\sum }}\frac{I_{i}}{4\pi \sigma r_{i}} \end{array}$$

Φ_*LFP*_ denotes the estimated LFP from transmembrane ionic currents.

As a second technique, LSA was implemented in LFPsim. LSA implementation assumed continuous distribution of the transmembrane currents generated from a line which passes through the axis of the neurite (see schematic in Figure 1D). LSA implementation have been known to better approximate the extracellular signal (except at very close distance, less than 1 μm away; Rosenfalck, 1969; Trayanova et al., 1990; Gold et al., 2006). LSA implementations have been previously modeled and validated on pyramidal neurons (Gold et al., 2006). Assuming line distribution of currents, extracellular potential of a line segment was estimated as

(5) $$\begin{array}{l} \Phi =\frac{1}{4\pi \sigma }\int_{-\Delta s}^{0}\frac{Ids}{\Delta s\sqrt{r^{2}+{\left(h-s\right)}^{2}}} \end{array}$$

(6) $$\begin{array}{l} \Phi =\frac{I}{4\pi \sigma \Delta s}\log\frac{\sqrt{h^{2}+r^{2}}-h}{\sqrt{l^{2}+r^{2}}-l} \end{array}$$

For “*n*” individual sources (line segments) in the extracellular medium, LFP was computed as in Equation (7),

(7) $$\begin{array}{l} \Phi_{LFP}=\overset{n\text{\_}sources}{\underset{i=1}{\sum }}\frac{I_{i}}{4\pi \sigma {△s}_{i}}\log\frac{\sqrt{{h_{i}}^{2}+{r_{i}}^{2}}-h_{i}}{\sqrt{{l_{i}}^{2}+{r_{i}}^{2}}-l_{i}} \end{array}$$

Where, Φ_*LFP*_ denotes the calculated LFP from transmembrane ionic current, “Δs” denotes the length of single line source, “*r*” denotes the radial distance from the line, “*h*” denotes the longitudinal distance from the end of the line, l = Δs+*h* denotes the distance from the start of the line, “σ” denotes the conductivity of the extracellular medium (see Figure 1D for schematic) and “*I*” denotes the transmembrane current generated from the source.

In another schema, neuronal processes were considered as passive compartments (Figure 1E). The low pass filtering property of the extracellular medium was modeled using a simple RC (low pass) filter (Bédard et al., 2004). Extracellular potential (Φ) at a distance “d” can be calculated as

(8) $$\begin{array}{l} \Phi =I\;e^{-\left(\frac{t}{E_{R}E_{C}}\right)} \end{array}$$

For “*n*” sources in extracellular medium, the computation is denoted as in Equation (9),

(9) $$\begin{array}{l} \Phi_{LFP}=\sum_{i=0}^{n\text{\_}sources}I_{i}e^{-\left(\frac{t}{E_{R}E_{C}}\right)} \end{array}$$

Where “V_0_” denotes individual compartment transmembrane ionic currents, “*t*” denotes the time constant (*t* = *E*_*r*_*E*_*c*_). “*E*_*C*_” denotes capacitance of extracellular medium, set to specific capacitance of the membrane (Johnston and Wu, 1995; Bédard et al., 2004). In this schema, homogenous capacitance was assumed throughout the extracellular space, “*E*_*R*_” denoted resistance of extracellular medium and standard value was assumed (cytoplasmic resistivity of squid axon set to 0.35 Ωm; see Bédard et al., 2006, for discussion on these parameters).

**Figure 2:**
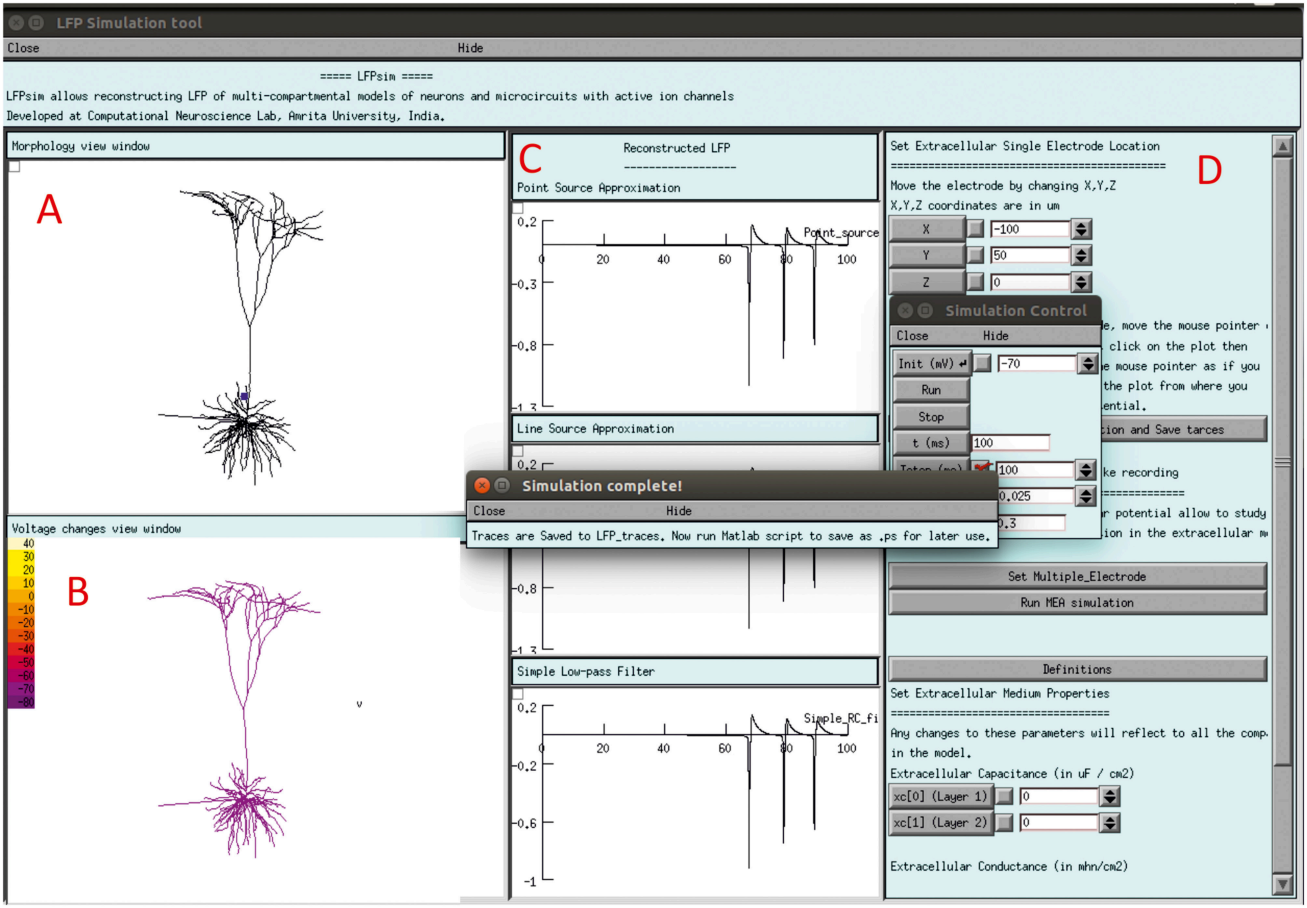
**LFPsim Graphical Interface. (A)** Morphology view window, **(B)** voltage changes view window, **(C)** computed LFP view panel, **(D)** simulation controls.

The LFPsim interface includes 4 views; Morphology view window (Figure 2A) is a shape plot which helps to visualize neuron morphology and extracellular electrode location (showed in blue). Right clicking on this window allows options to the user to view the model at different angles and allows selecting LFP electrode location by selecting “LFP_electrode” menu, Voltage changes view window (Figure 2B) is a space plot to visualize voltage changes across the neuronal membrane during activity. Voltage range from −70 to +40 mV was defined as color map; computed LFP view panel (Figure 2C) allows viewing the simulated LFP trace in all three methods; simulation control (Figure 2D) includes controls for setting electrode parameters and extracellular medium properties and running simulation. LFPsim will be made publically available at ModelDB (http://modeldb.yale.edu).

### Computing MEA field potential

**Figure 3:**
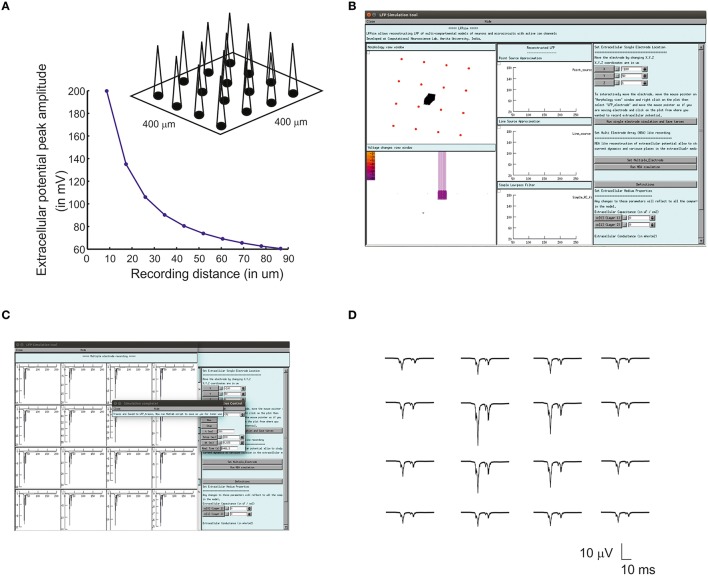
**Simulated “Multi Electrode Array” of evoked cerebellar granular layer post-synaptic LFP**. **(A)** Schematic illustration of Multi Electrode Array (MEA) electrode and spatial attenuation N_2a_ cerebellar LFP waves. **(B)** Screenshot of LFPsim MEA simulation on cerebellar microcircuit. **(C,D)** Computed MEA LFP. **(D)** Variations in center-surround activity showed augmented width and amplitude in simulated LFP from the center electrodes compared to the periphery.

Multiple Electrode Array (MEA) recordings have been extensively used in electrophysiology to study neuronal circuit function in brain slices and intact brains (BeMent et al., 1986; Mapelli and D'Angelo, 2007; Mapelli et al., 2010; Spira and Hai, 2013). Computational reconstruction of multiple electrodes was aimed to help observe, constrain and model activity at network level. In LFPsim, “MEA” like observation was modeled mathematically by spatially arranging individual electrodes in a square matrix (see Figures 1C, 3A). The tip of the individual electrode was considered as the recording point of the electrodes. The effect of saline layer interfacing between brain slice and MEA chip is not considered in the model in order to reduce the complexity (Ness et al., 2015). Field potential generated from a neuronal area was modeled using LSA (see Equations 6, 7). LFP at a point, *MEA(x,y,z)* was computed using Equation (10).

(10) $$\begin{array}{l} MEA\left(x,y,z\right)=\Phi_{LFP}\left(x,y,z\right) \end{array}$$

For multi-electrode computation, a cerebellar granular layer network model was used to reproduce “MEA” LFP feature of LFPsim. The network model was simulated with center-surround excitation (Parasuram et al., 2015), with a single spike as input (*in vitro* behavior) through mossy fibers. The cerebellar circuit was simulated for 200 ms, input stimuli to network was provided at *t* = 20 ms through mossy fibers by single spike to compute *in vitro* post-synaptic LFP (Mapelli and D'Angelo, 2007; Diwakar et al., 2011; Parasuram et al., 2011). After loading the model in LFPsim in NEURON, multi electrode location was set by accessing “MEA” properties described in LFPsim (see Figures 3B,C). By default, an array of 4-by-4 LFP electrodes was deployed and distance between electrodes was set to 100 microns (see schematic, Figure 3A). The number of electrodes, distance and plane of electrode can be altered via the LFPsim interface.

### Models of neurons and networks

The cerebellum granule neuron model (Diwakar et al., 2009), a detailed biophysical model consisting of 52 active cable compartments, was used to compute extracellular potential. Distribution and localization of the channels have been described previously (D'Angelo et al., 2001; Diwakar et al., 2009). The model has four excitatory (with AMPA and NMDA receptor dynamics) and four inhibitory (GABA) synapses, located at the distal end of the dendrites. A mossy fiber excites both Granule and Golgi cells of same glomeruli. In the model, the mossy fibers excited granule cells via excitatory synapses (D'Angelo et al., 2001; Nieus et al., 2006) and Golgi cell axon inhibited the granule cells via GABAergic synapse (Nieus et al., 2006).

A detailed biophysical model of the cerebellum Golgi neuron (Solinas et al., 2007) was used to construct the cerebellar network. The Golgi neuron model consisted of a soma, three dendrites and an axon. Most of the ion channels mechanisms were placed at the soma as described in Solinas et al. (2007). Synaptic model including AMPA, NMDA, GABA receptor dynamics were implemented as described in Solinas et al. (2010).

Pyramidal neuron models were adapted from Mainen and Sejnowski (1996), models of L3 Pyramidal neuron, L3 Stellate neuron and L5 Pyramidal neuron were used to study extracellular field potential computation in complex neurons. The models were stimulated by somatic current injection 70, 100, 200 pA and synaptic activations as reported in Mainen and Sejnowski (1996).

The cerebellar granular layer network model consisted of 730 Multi-compartmental granule neurons (Diwakar et al., 2009), 40 Mossy Fibers (MF) and approximately 8500 synapses to a pack 35 μm cubic slice of cerebellar cortex. The network was simulated with *in vitro* pattern (single spike as input at 500 Hz) to generate the N_2a_ and N_2b_ waves (**Figure 5A**) as reported in Mapelli and D'Angelo (2007) and in simulation by Diwakar et al. (2011) and Parasuram et al. (2011). Plasticity conditions were simulated in cerebellar granular layer by modifying intrinsic excitability of sodium channel and release probability of excitatory synapses (Nieus et al., 2006).

As an alternative model in this study, a biophysical network model of neocortex (Vierling-Claassen et al., 2010) was used, (ModelDB accession no: 141273). The model represents cortical layer II/III circuits containing three cell types: pyramidal fast spiking, regular spiking, and low-threshold spiking interneurons. The model was developed in NEURON and input to the model was varied at frequencies to generate LFP waves at 8, 32, and 56 Hz FS as mentioned in Vierling-Claassen et al. (2010).

### Implementation

LFP modeling schemas (PSA, LSA, and RC methods) were implemented in “*extracellular_electrode.hoc.”* In LFPsim, field potential calculation at each time step, dt, was coded in NMODL, “*lfp.mod*” was for simulating single electrode LFP simulation and “*mea.mod*” for multiple electrode simulation. Procedures for changing electrode recording position were implemented in “*move_electrode.hoc.”* “*multiple_electrode.hoc”* contains implementation of LFP for multiple electrode simulation. “*multiple_electrode1.hoc”* contains functions to set multi electrode simulation. “*mea_run_then_plot.hoc,”* contains the functions to plot MEA trace using a NEURON graph plot and “*tool_interface.hoc”* contains the GUI interface script. Details for using LFPsim for computing single extracellular potential and population LFP are included in Supplementary Material. The current version of the tool was not designed to run on parallel platforms, and therefore may not be suitable for very large scale networks.

### Simulations

All simulations were performed with the NEURON version 7.4 simulation environment. Single neuron and network simulations were performed on a 6-core Intel(R) Xeon(R) CPU W3670 at 3.20 GHz processor and 8GB of RAM. All code is available from ModelDB (http://modeldb.yale.edu) with accession number 190140.

## Results

### Computing single neuron electric potentials depend on detailed biophysical modeling

Using LFPsim, extracellular field potentials from electrotonically compact cerebellar granule neuron (Diwakar et al., 2009) and morphologically complex neurons of neocortical column (Mainen and Sejnowski, 1996) were computed.

**Figure 4:**
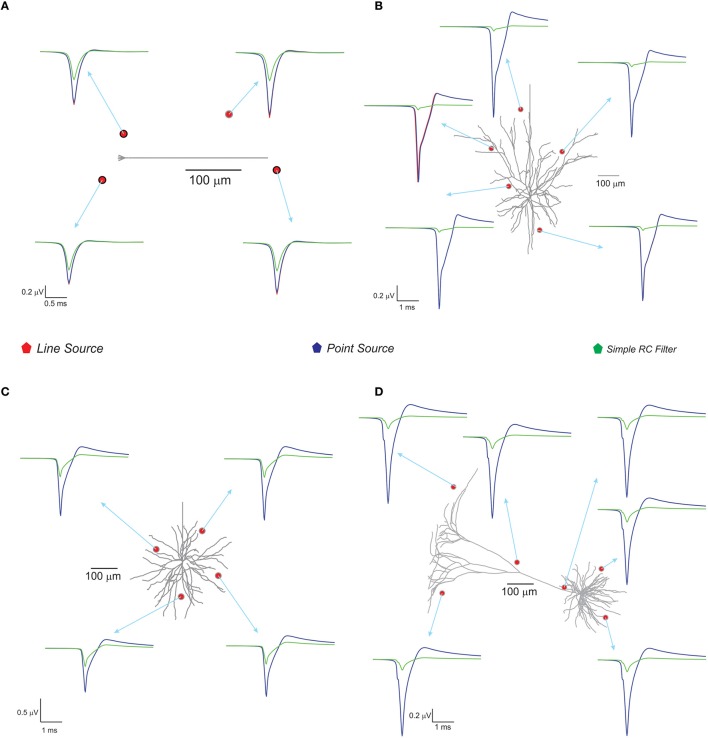
**Computed single neuron electric potential and comparison of methods in electrotonically compact and morphologically complex neurons**. **(A)** Cerebellum granule cell model (Diwakar et al., 2009) and computed single neuron evoked LFP when 3 excitatory inputs via mossy fibers were simulated. The extracellular electrode near to soma showed increased amplitude to extracellular wave compare to other recording position around the neuron. **(B–D)** Show LFP related to neuronal models of neocortical column. **(B)** L3 pyramidal neuron model, **(C)** L3 stellate neuron model, and **(D)** L5 pyramidal neuron model. Neocortical models were evoked by somatic current injection (70, 100, 200 pA for **B–D**, respectively; Mainen and Sejnowski, 1996) and the corresponding extracellular potential for the first spike was calculated and plot for various locations in the neighborhood of the neuron. Red, blue, green traces were extracellular signals estimated using LSA, PSA, and low pass filter based techniques respectively.

With spike inputs via 3 mossy fiber synapses, the granule neuron model generated a single action potential. Extracellular potential calculated at a point in the vicinity of the axon showed increased amplitude compared to other recording positions near the neuron (see Figure 4). The techniques, PSA and LSA approximations showed similar responses in terms of estimated extracellular signal for single neurons. The low pass filter based method exhibits reduced amplitude and width in the computed wave (see Figure 4). The nature of the single neuron extracellular potential depended on compartmental contributions and morphological details (Parasuram et al., 2011). We also observed that non-detailed models did not provide reliable field potential reconstructions (data not shown).

With the neocortical column (Mainen and Sejnowski, 1996), L3 pyramidal neuron, L3 stellate neuron, and L5 pyramidal neuron models, extracellular potential computations were similar to those in experimental studies. Somatic current injections (70–200 pA) were provided as inputs (Mainen and Sejnowski, 1996) and extracellular potential for the first spike was calculated (see Figure 4). The RC-based method showed reduced extracellular signal amplitude and width (See Figure 4) although PSA and LSA had no significant differences.

### Estimating local field potential from microcircuits

In this study, two biophysically detailed network models, implemented in NEURON were used to compute network LFPs. For structurally less complex, small scale networks, the cerebellar granular layer model was used (Parasuram et al., 2011). A network model of neocortex (Vierling-Claassen et al., 2010) was used to investigate the role of cable structures in LFP simulations on complex microcircuit models.

#### Cerebellar granular layer evoked LFP reconstructions showed N_2_ waves *IN vitro* and T-C waves *in vivo*

**Figure 5:**
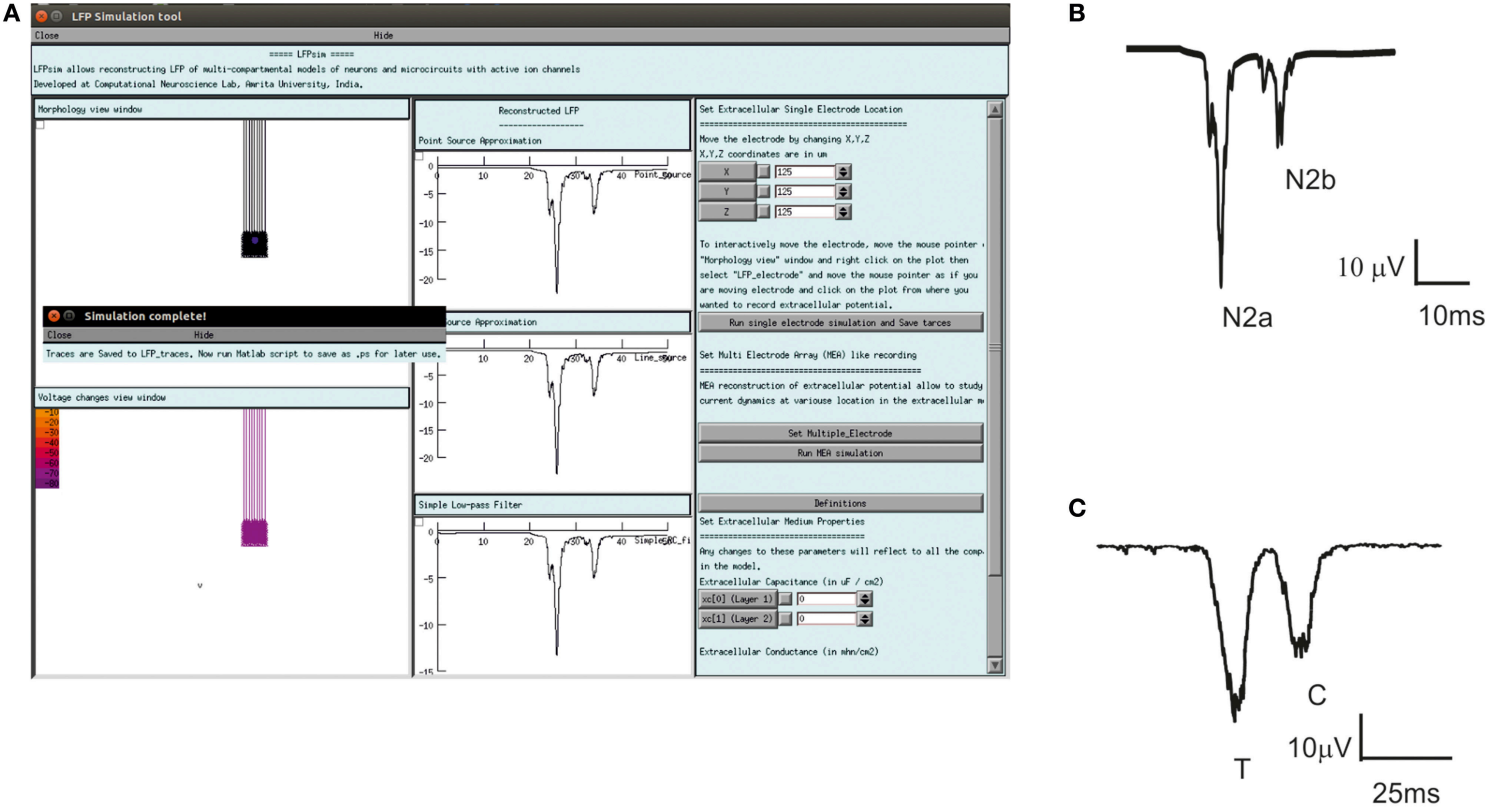
**Computed evoked ***in vitro***, ***in vivo*** LFP waves of cerebellum granular layer using LFPsim**. **(A)** Cerebellar granular layer network in LFPsim. **(B)** Computed *in vitro* LFP generated negative waves corresponding to the first spike (N_2a_) and doublet (N_2b_). **(C)** Simulated *in vivo* LFP showed trigeminal (T) and cortical **(C)** waves.

With single spikes as mossy fiber inputs, evoked post-synaptic LFP on cerebellum granular layer generated the N_2a_ and N_2b_ waves (Mapelli and D'Angelo, 2007; see Figure 5B). With bursts as inputs to reflect trigeminal and cortical inputs via mossy fibers, *in vivo* evoked LFP simulations generated T and C waves as observed in Crus-IIa (Bower and Woolston, 1983) of rat cerebellum. Computed *in vivo* LFP (see Figure 5C) also reproduced plasticity related amplitude and lag changes (Diwakar et al., 2011; Parasuram et al., 2011).

#### Simulating distinct neocortical oscillations reproduce enhanced LFP

**Figure 6:**
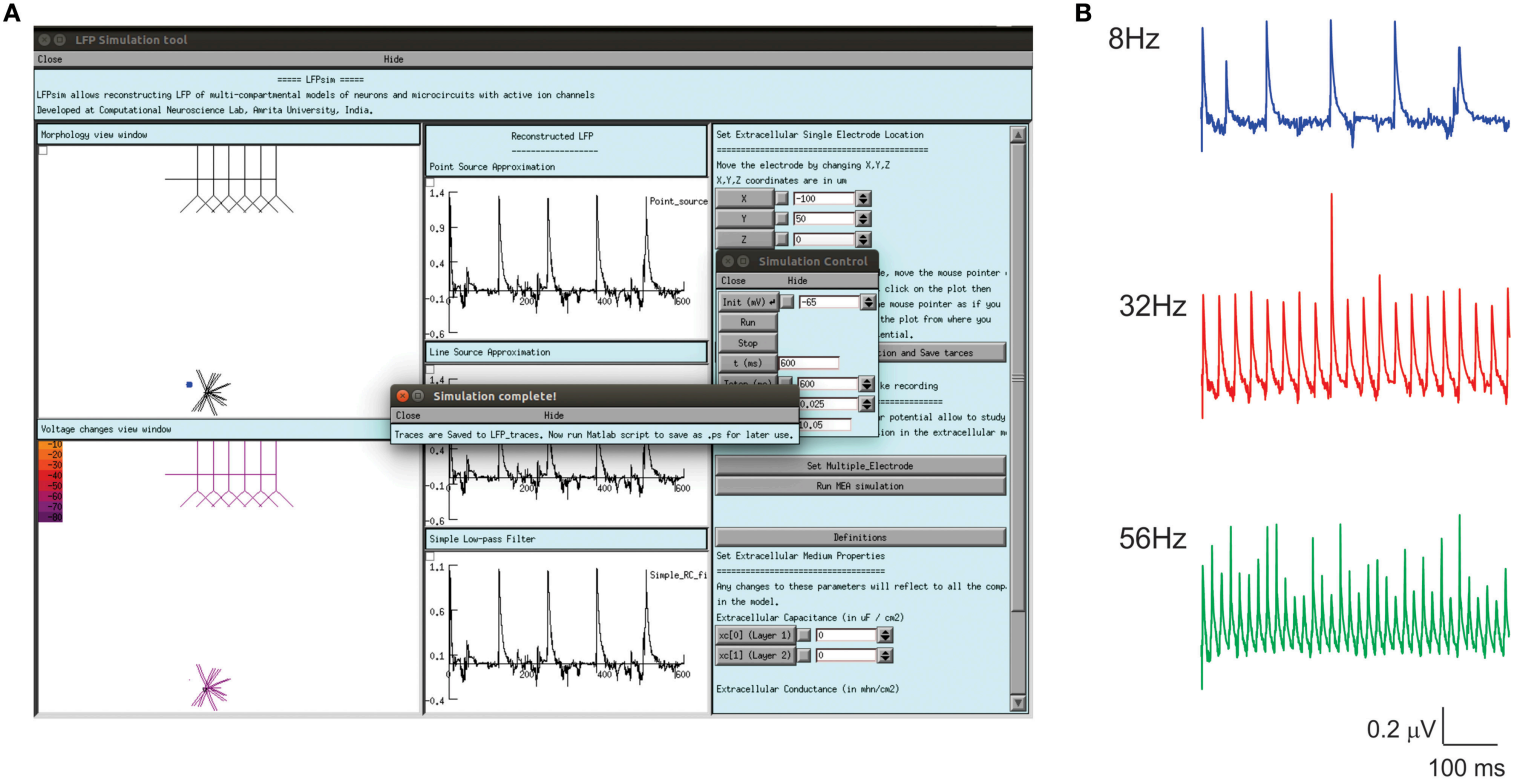
**Modeled evoked LFP of neocortical microcircuit using LFPsim**. **(A)** Neocortical microcircuit and computed 8 Hz LFP wave using LFPsim. **(B)** Simulated LFP at 8, 32, and 56 Hz (see also Figure 3C of Vierling-Claassen et al., 2010).

Enhanced LFP by regular spiking interneurons in primary somatosensory neocortex has been known to be controlled by low-threshold spiking and fast-spiking interneurons (Vierling-Claassen et al., 2010). On the neocortical microcircuit simulations with LFPsim, gamma resonance during FS drive was reproduced (see Figures 6A,B) as reported in the study (Vierling-Claassen et al., 2010). Light drive input frequency in the model was varied at frequencies (8, 32, and 56 Hz) to generate corresponding LFP wave responses.

### Comparison of different modeling schemas and their computational complexity

**Figure 7:**
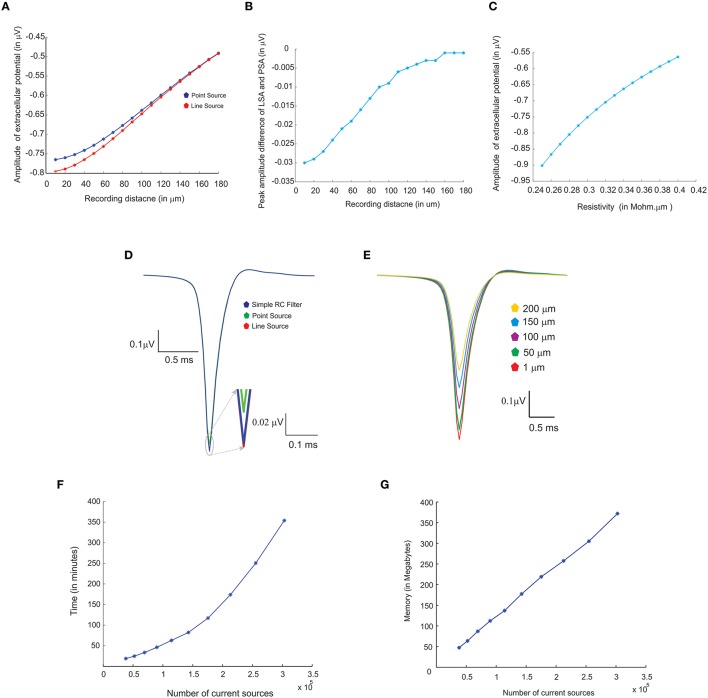
**Comparing different modeling schema for calculating extracellular potential. (A)** Comparison of amplitudes of extracellular potential calculated using LSA and PSA of a single granule cell model (Diwakar et al., 2009). **(B)** Amplitude difference between LSA and PSA with increasing recording distance. **(C)** Variations in extracellular potential amplitude by increasing the extracellular resistivity from 0.24 to 0.42 Ωm (Nicholson and Freeman, 1975; Goto et al., 2010; Einevoll et al., 2013). **(D)** Comparing LSA, PSA, and RC-filter methods by computing extracellular waveforms of a single granule neuron model (Diwakar et al., 2009). **(E)** Attenuation of extracellular potential with increase in recording distance. **(F)** Simulation time for increasing number of current sources to evaluate computational load. **(G)** RAM usage for increasing number of current sources.

Three modeling methods generated extracellular field potentials of single neurons and neural microcircuits. Computed extracellular waves (based on PSA and LSA) showed linear drop in amplitude when the recording electrode moved away from the neuronal process (see Figures 7A,E). LFP wave computed from (PSA and LSA) also showed the similar amplitude and shape for recording sites farther than 120 microns from the neurite (see Figure 7B).

Extracellular potentials of a granule neuron, computed when the electrode was in the vicinity to the soma (Figure 7D, LSA in red, PSA in green, Simple RC in blue) showed similar behavior. LSA and RC based LFP waveforms showed similar amplitude but the PSA generated LFP wave showed slightly reduced amplitude compared to the other techniques.

Attenuation properties of the extracellular medium was studied on a multi compartmental model of the granule neuron (Diwakar et al., 2009) by varying extracellular resistivity from 0.24 to 0.42 Ωm (Nicholson and Freeman, 1975; Goto et al., 2010). Extracellular wave amplitude showed a linear decrease, when the resistivity of extracellular medium increased (see Figure 7C).

LFP algorithmic implementations were analyzed using RAM model of order analysis (Hartmanis, 1971). PSA-based estimations of extracellular electric potential of a single current source at a given point for a single time step (dt) used in 21 unit operations (each arithmetic operation was counted as one unit in order analysis methods), LSA used 70 units and RC filter used 21 units. For a neuron model with “*n*” compartments, 70^*^n units for LSA to calculate the electric potential for single time step. For large “*n*” on network models, the algorithms will run in the order of TS, O (TS) where “TS,” denotes the total time steps in the simulation.

Computation time and memory requirements to simulate large scale network models of LFPsim was analyzed. Real time measurements for computed LFPs were plotted corresponding to a 100 ms of simulation (See Figure 7F). Augmenting granule neuron population sizes indicated a linear increment in computing time when network model was scaled by increasing number of current sources (Figure 7F). The amount of RAM used to compute LFP for a model did not vary much throughout the simulation (Figure 7G).

### Estimating multiple electrode field potential using LFPsim

Using multiple spaced virtual recording electrodes, *in vitro* “MEA” recording of granular layer and spatial attenuation of cerebellar N_2a_ and N_2b_ LFP waves (see Figure 3D) was simulated in a 400 × 400 μm area of granule neurons with 100 μm spacing among electrodes. LFP computed from peripheral electrodes showed decreased amplitude and width of N_2a_ and N_2b_ waves (Diwakar et al., 2011) in comparison to the center of MEA (Figure 3D). Array-based representation also reproduced the inherent amplitude decay modeled as a characteristic of the extracellular space.

## Discussion

In this paper, we have presented LFPsim as a NEURON-based script, to mathematically compute single neuron electric potential and population LFP. LFPsim uses the forward modeling method to model extracellular potential. Three biophysical modeling schemas, PSA, LSA (Holt and Koch, 1999; Gold et al., 2006) and RC-based low pass filter techniques were implemented to model the extracellular activity. LFPsim uses NEURON to execute the algorithms, the schemas were implemented in hoc and NMODL (Hines and Carnevale, 1997).

We were able to compute LFP from several biophysical models of neurons and networks implemented in NEURON. For single neuron electric potential computations, we chose an electrically compact neuron, cerebellar granule neuron (Diwakar et al., 2009) and a structurally complex, pyramidal neuron (Mainen and Sejnowski, 1996). A simulation on single compartmental models exhibited unreliable LFPs compared to that of multicompartmental models (Parasuram et al., 2011). To test the reliability of LFPsim in computing LFP on microcircuits we used a neocortical network (Vierling-Claassen et al., 2010) and cerebellar granule cell population. LFPsim reproduced *in vitro* N_2a_ and N_2b_ post-synaptic LFP waves of cerebellar granular layer and neocortical LFP's at 8, 32, and 56 Hz (current injection). The tool also allowed network models to be recruited for “multi-electrode array” like LFP traces and to allow model-based testing of spatial attenuation properties of extracellular medium.

In addition to mechanistic properties related to extracellular spike time, LFPsim also computed cerebellum granular layer evoked LFP during induced plasticity conditions, LTD and LTP. LTD showed the depression of width and lesser amplitude while LTP shows bigger amplitude and wider wave width in the T–C (Trigeminal and Cortical) components of the *in vivo* waveform. LFPsim was also employed on other biophysical neuron and network models (Ferrante et al., 2008; Morgan and Soltesz, 2008; Hu et al., 2009; Publio et al., 2009; Li and Cleland, 2013).

Simulations indicated that extracellular potentials generated using PSA and LSA showed little difference in amplitude and width for a distant neurite (>120 μm) from the recording point. But, simulations of LFPs in close proximity to neurons on both single neurons and network models suggested that the LSA technique was more accurate for morphologically complex neurons and on electronically compact neurons like granule neurons. The role of extracellular resistivity on the nature of the extracellular field potential was studied by simulating field potential by varying the resistance from 0.24 to 0.42 MΩ-mm consistent with the coupled role of extracellular medium attenuation on decreases of recorded wave amplitude.

On-the-fly implementation of LFP modeling schemas without disk write, effectively reduced I/O operations and the need for additional secondary storage (Figure 7G). We also compared the implementation with an offline implementation where we saved all ionic currents as a plain text file. The simulations of LFPs from stored ionic currents required a few gigabytes of storage and 16 min of runtime on a workstation with 6 CPU cores running at 3.20 GHz processor and 8GB of RAM. On-the-fly estimation also helps to compute cortical LFP without saving the transmembrane currents into additional storage (Glabska et al., 2014). Additionally, we employed NEURON's Interviews library components for the GUI. In this version, all electrode parameters and the extracellular medium properties can be directly accessed via the interface.

A variety of LFP modeling tools available today, study origin and nature of extracellular potential calculated from multicompartmental neuron models. Python-based LFPy (Lindén et al., 2014), ViSAPy, that extends LFPy for calculating extracellular potential from multicompartmental neuron models and for any geometries of recording electrodes (Hagen et al., 2015), ViMEAPy, a python-based Multi Electrode Array (MEA) trace modeling tool (Ness et al., 2015) and VERTEX (Tomsett et al., 2015) were different from LFPsim in terms of use with NEURON (Hines and Carnevale, 1997) hoc models. Compared to LFPy, VERTEX, ViSAPy, and ViMEAPy, LFPsim computes LFPs from NEURON hoc models allowing some of the ModelDB NEURON models to be used without significant re-implementation. In LFPsim, MEA like recording was implemented as a computation of LFPs arranged to seem like a spatial matrix. Unlike in ViMEAPy, the effect of saline bath was not implemented in the tool (Hagen et al., 2015). In our simulations, LFPsim could compute LFP from large network models composed of a million current sources. Although, we foresee a future implementation on python-based models and for MPI-compatible simulations, LFPsim is currently unusable with parallel models.

## Conclusion

LFPsim provides a tool-enabled approach to study extracellular potential of single cells and small neural populations. The current version of LFPsim can be employed on existing NEURON models to compute extracellular field potential in single neurons and network implementations. LFPsim is publicly available on ModelDB (https://senselab.med.yale.edu/ModelDB/ShowModel.cshtml?model=190140).

## Author contributions

HP, SD implemented the LFP code and performed all the simulations in this manuscript. HP, BN, MH, ED, GN, and SD contributed in interpretation of simulations. HP, BN, ED, MH, GN, and SD contributed to conception and design of the work and in developing the manuscript.

### Conflict of interest statement

The authors declare that the research was conducted in the absence of any commercial or financial relationships that could be construed as a potential conflict of interest.

## References

[B1] Bédard C. Kröger H. Destexhe A. (2004). Modeling extracellular field potentials and the frequency-filtering properties of extracellular space. Biophys. J. 86, 1829–1842. 10.1016/S0006-3495(04)74250-2 14990509 PMC1304017

[B2] Bédard C. Kröger H. Destexhe A. (2006). Model of low-pass filtering of local field potentials in brain tissue. Phys. Rev. E Stat. Nonlin. Soft Matter Phys. 73:051911. 10.1103/PhysRevE.73.051911 16802971

[B3] BeMent S. L. Wise K. D. Anderson D. J. Najafi K. Drake K. L. (1986). Solid-state electrodes for multichannel multiplexed intracortical neuronal recording. IEEE Trans. Biomed. Eng. 33, 230–241. 10.1109/TBME.1986.325895 3957372

[B4] Bower J. M. Woolston D. C. (1983). Congruence of spatial organization of tactile projections to granule cell and Purkinje cell layers of cerebellar hemispheres of the albino rat: vertical organization of cerebellar cortex. J. Neurophysiol. 49, 745–766. 6300353 10.1152/jn.1983.49.3.745

[B5] Buzsaki G. (2006). Rhythms of the Brain. Oxford: Oxford University Press.

[B6] Buzsáki G. (2004). Large-scale recording of neuronal ensembles. Nat. Neurosci. 7, 446–451. 10.1038/nn1233 15114356

[B7] Buzsáki G. Anastassiou C. A. Koch C. (2012). The origin of extracellular fields and currents–EEG, ECoG, LFP and spikes. Nat. Rev. Neurosci. 13, 407–420. 10.1038/nrn3241 22595786 PMC4907333

[B8] Caton R. (1874). The electric currents of the brain. Br. Med. J. 2, 265–278.

[B9] Colgin L. L. Denninger T. Fyhn M. Hafting T. Bonnevie T. Jensen O.. (2009). Frequency of gamma oscillations routes flow of information in the hippocampus. Nature 462, 353–357. 10.1038/nature08573 19924214

[B10] D'Angelo E. Nieus T. Maffei A. Armano S. Rossi P. Taglietti V.. (2001). Theta-frequency bursting and resonance in cerebellar granule cells: experimental evidence and modeling of a slow k^+^-dependent mechanism. J. Neurosci. 21, 759–770. 11157062 10.1523/JNEUROSCI.21-03-00759.2001 PMC6762330

[B11] De Schutter E. (2010). Computational Modeling Methods for Neuroscientists. Cambridge, MA: MIT Press.

[B12] Di S. Baumgartner C. Barth D. S. (1990). Laminar analysis of extracellular field potentials in rat vibrissa/barrel cortex. J. Neurophysiol. 63, 832–840. 2341880 10.1152/jn.1990.63.4.832

[B13] Diwakar S. Lombardo P. Solinas S. Naldi G. D'Angelo E. (2011). Local field potential modeling predicts dense activation in cerebellar granule cells clusters under LTP and LTD control. PLoS ONE 6:e21928. 10.1371/journal.pone.0021928 21818278 PMC3139583

[B14] Diwakar S. Magistretti J. Goldfarb M. Naldi G. D'Angelo E. (2009). Axonal Na+ channels ensure fast spike activation and back-propagation in cerebellar granule cells. J. Neurophysiol. 101, 519–532. 10.1152/jn.90382.2008 19073816 PMC2657054

[B15] Eccles J. C. (1951). Interpretation of action potentials evoked in the cerebral cortex. Electroencephalogr. Clin. Neurophysiol. 3, 449–464. 10.1016/0013-4694(51)90033-8 14887631

[B16] Egert U. Heck D. Aertsen A. (2002). Two-dimensional monitoring of spiking networks in acute brain slices. Exp. Brain Res. 142, 268–274. 10.1007/s00221-001-0932-5 11807580

[B17] Einevoll G. T. Kayser C. Logothetis N. K. Panzeri S. (2013). Modelling and analysis of local field potentials for studying the function of cortical circuits. Nat. Rev. Neurosci. 14, 770–785. 10.1038/nrn3599 24135696

[B18] Ferrante M. Blackwell K. T. Migliore M. Ascoli G. A. (2008). Computational models of neuronal biophysics and the characterization of potential neuropharmacological targets. Curr. Med. Chem. 15, 2456–2471. 10.2174/092986708785909094 18855673 PMC3560392

[B19] Glabska H. Potworowski J. Leski S. Wojcik D. K. (2014). Independent components of neural activity carry information on individual populations. PLoS ONE 9:e105071. 10.1371/journal.pone.0105071 25153730 PMC4143226

[B20] Gleeson P. Piasini E. Crook S. Cannon R. Steuber V. Jaeger D. (2012). The open source brain initiative: enabling collaborative modelling in computational neuroscience. BMC Neurosci. 13(Suppl. 1):O7 10.1186/1471-2202-13-S1-O7

[B21] Gold C. Henze D. A. Koch C. (2007). Using extracellular action potential recordings to constrain compartmental models. J. Comput. Neurosci. 23, 39–58. 10.1007/s10827-006-0018-2 17273940

[B22] Gold C. Henze D. A. Koch C. Buzsáki G. Buzsaki G. (2006). On the origin of the extracellular action potential waveform: a modeling study. J. Neurophysiol. 95, 3113–3128. 10.1152/jn.00979.2005 16467426

[B23] Goto T. Hatanaka R. Ogawa T. Sumiyoshi A. Riera J. Kawashima R. (2010). An evaluation of the conductivity profile in the somatosensory barrel cortex of Wistar rats. J. Neurophysiol. 104, 3388–3412. 10.1152/jn.00122.2010 20810682

[B24] Hagen E. Ness T. V. Khosrowshahi A. Sørensen C. Fyhn M. Hafting T.. (2015). ViSAPy: a Python tool for biophysics-based generation of virtual spiking activity for evaluation of spike-sorting algorithms. J. Neurosci. Methods 245, 182–204. 10.1016/j.jneumeth.2015.01.029 25662445

[B25] Hämäläinen M. Hari R. Ilmoniemi R. J. Knuutila J. Lounasmaa O. V. (1993). Magnetoencephalography—theory, instrumentation, and applications to noninvasive studies of the working human brain. Rev. Mod. Phys. 65, 413–497. 10.1103/RevModPhys.65.413

[B26] Hartmanis J. (1971). Computational complexity of random access stored program machines. Math. Syst. Theory 5, 232–245. 10.1007/BF01694180

[B27] Hines M. L. Carnevale N. T. (1997). The NEURON simulation environment. Neural Comput. 9, 1179–1209. 9248061 10.1162/neco.1997.9.6.1179

[B28] Hines M. L. Carnevale N. T. (2000). Expanding {NEURON}'s repertoire of mechanisms with {NMODL}. Neural Comput. 12, 995–1007. 10.1162/089976600300015475 10905805

[B29] Hines M. L. Morse T. Migliore M. Carnevale N. T. Shepherd G. M. (2004). ModelDB: a database to support computational neuroscience. J. Comput. Neurosci. 17, 7–11. 10.1023/B:JCNS.0000023869.22017.2e 15218350 PMC3732827

[B30] Holt G. (1998). A Critical Reexamination of Some Assumptions and Implications of Cable Theory in Neurobiology. Ph.D. thesis, California Institute of Technology, Pasadena, CA.

[B31] Holt G. R. Koch C. (1999). Electrical interactions via the extracellular potential near cell bodies. J. Comput. Neurosci. 6, 169–184. 10333161 10.1023/a:1008832702585

[B32] Hu W. Tian C. Li T. Yang M. Hou H. Shu Y. (2009). Distinct contributions of Na(v)1.6 and Na(v)1.2 in action potential initiation and backpropagation. Nat. Neurosci. 12, 996–1002. 10.1038/nn.2359 19633666

[B33] Johnston D. Wu S. M. S. (1995). Foundations of Cellular Neurophysiology. Cambridge, MA: MIT Press.

[B34] Kandel A. Buzsáki G. (1997). Cellular-synaptic generation of sleep spindles, spike-and-wave discharges, and evoked thalamocortical responses in the neocortex of the rat. J. Neurosci. 17, 6783–6797. 9254689 10.1523/JNEUROSCI.17-17-06783.1997 PMC6573130

[B35] Li G. Cleland T. A. (2013). A two-layer biophysical model of cholinergic neuromodulation in olfactory bulb. J. Neurosci. 33, 3037–3058. 10.1523/JNEUROSCI.2831-12.2013 23407960 PMC3711624

[B36] Lindén H. Hagen E. £êski S. Norheim E. S. Pettersen K. H. Einevoll G. T. (2014). LFPy: a tool for biophysical simulation of extracellular potentials generated by detailed model neurons. Front. Neuroinform. 7:41. 10.3389/fninf.2013.00041 24474916 PMC3893572

[B37] Lindén H. Tetzlaff T. Potjans T. C. Pettersen K. H. Grün S. Diesmann M.. (2011). Modeling the spatial reach of the LFP. Neuron 72, 859–872. 10.1016/j.neuron.2011.11.006 22153380

[B38] Mainen Z. F. Sejnowski T. J. (1996). Influence of dendritic structure on firing pattern in model neocortical neurons. Nature 382, 363–366. 10.1038/382363a0 8684467

[B39] Mapelli J. D'Angelo E. (2007). The spatial organization of long-term synaptic plasticity at the input stage of cerebellum. J. Neurosci. 27, 1285–1296. 10.1523/JNEUROSCI.4873-06.2007 17287503 PMC6673576

[B40] Mapelli J. Gandolfi D. D'Angelo E. (2010). Combinatorial responses controlled by synaptic inhibition in the cerebellum granular layer. J. Neurophysiol. 103, 250–261. 10.1152/jn.00642.2009 19906881

[B41] Mehring C. Rickert J. Vaadia E. Cardosa de Oliveira S. Aertsen A. Rotter S. (2003). Inference of hand movements from local field potentials in monkey motor cortex. Nat. Neurosci. 6, 1253–1254. 10.1038/nn1158 14634657

[B42] Migliore M. Morse T. M. Davison A. P. Marenco L. Shepherd G. M. Hines M. L. (2003). ModelDB: making models publicly accessible to support computational neuroscience. Neuroinformatics 1, 135–139. 10.1385/NI:1:1:135 15055399 PMC3728921

[B43] Mineault P. J. Zanos T. P. Pack C. C. (2013). Local field potentials reflect multiple spatial scales in V4. Front. Comput. Neurosci. 7:21. 10.3389/fncom.2013.00021 23533106 PMC3607798

[B44] Mitzdorf U. (1985). Current source-density method and application in cat cerebral cortex: investigation of evoked potentials and EEG phenomena. Physiol. Rev. 65, 37–100. 3880898 10.1152/physrev.1985.65.1.37

[B45] Montgomery S. M. Buzsáki G. (2007). Gamma oscillations dynamically couple hippocampal CA3 and CA1 regions during memory task performance. Proc. Natl. Acad. Sci. U.S.A. 104, 14495–14500. 10.1073/pnas.0701826104 17726109 PMC1964875

[B46] Morgan R. J. Soltesz I. (2008). Nonrandom connectivity of the epileptic dentate gyrus predicts a major role for neuronal hubs in seizures. Proc. Natl. Acad. Sci. U.S.A. 105, 6179–6184. 10.1073/pnas.0801372105 18375756 PMC2299224

[B47] Ness T. V. Chintaluri C. Potworowski J. Łęski S. Głąbska H. Wójcik D. K.. (2015). Modelling and analysis of electrical potentials recorded in microelectrode arrays (MEAs). Neuroinformatics 13, 403–426. 10.1007/s12021-015-9265-6 25822810 PMC4626530

[B48] Nicholson C. Freeman J. A. (1975). Theory of current source-density analysis and determination of conductivity tensor for anuran cerebellum. J. Neurophysiol. 38, 356–368. 805215 10.1152/jn.1975.38.2.356

[B49] Nieus T. Sola E. Mapelli J. Saftenku E. Rossi P. Angelo E. D. (2006). LTP regulates burst initiation and frequency at mossy fiber–Granule Cell Synapses of Rat Cerebellum: experimental observations and theoretical predictions. J. Neurophysiol. 95, 686–699. 10.1152/jn.00696.2005 16207782

[B50] Parasuram H. Nair B. Naldi G. Diwakar S. D'Angelo E. (2011). A modeling based study on the origin and nature of evoked post-synaptic local field potentials in granular layer. J. Physiol. Paris 105, 71–82. 10.1016/j.jphysparis.2011.07.011 21843640

[B51] Parasuram H. Nair B. Naldi G. D'Angelo E. Diwakar S. (2015). Exploiting point source approximation on detailed neuronal models to reconstruct single neuron electric field and population LFP, in 2015 International Joint Conference on Neural Networks (IJCNN) (Killarney: IEEE), 1–7. 10.1109/IJCNN.2015.7280607

[B52] Plonsey R. (1969). Bioelectric Phenomena. New York, NY: McGraw-Hill.

[B53] Publio R. Oliveira R. F. Roque A. C. (2009). A computational study on the role of gap junctions and rod Ih conductance in the enhancement of the dynamic range of the retina. PLoS ONE 4:e6970. 10.1371/journal.pone.0006970 19777063 PMC2745074

[B54] Rall W. Shepherd G. M. (1968). Theoretical reconstruction of field potentials and dendrodendritic synaptic interactions in olfactory bulb. J. Neurophysiol. 31, 884–915. 5710539 10.1152/jn.1968.31.6.884

[B55] Reimann M. W. Anastassiou C. A. Perin R. Hill S. L. Markram H. Koch C. (2013). A biophysically detailed model of neocortical local field potentials predicts the critical role of active membrane currents. Neuron 79, 375–390. 10.1016/j.neuron.2013.05.023 23889937 PMC3732581

[B56] Rickert J. Oliveira S. C. de Vaadia E. Aertsen A. Rotter S. Mehring C. (2005). Encoding of movement direction in different frequency ranges of motor cortical local field potentials. J. Neurosci. 25, 8815–8824. 10.1523/JNEUROSCI.0816-05.2005 16192371 PMC6725584

[B57] Rosenfalck P. (1969). Intra- and extracellular potential fields of active nerve and muscle fibres. A physico-mathematical analysis of different models. Acta Physiol. Scand. Suppl. 321, 1–168. 5383732

[B58] Scherberger H. Jarvis M. R. Andersen R. A. (2005). Cortical local field potential encodes movement intentions in the posterior parietal cortex. Neuron 46, 347–354. 10.1016/j.neuron.2005.03.004 15848811

[B59] Segev I. Fleshman J. W. Burke R. E. (1989). Compartmental models of complex neurons, in Methods in Neuronal Modeling, eds Koch C. Segev I. (Cambridge, MA: MIT Press), 63–96.

[B60] Solinas S. Forti L. Cesana E. Mapelli J. De Schutter E. D'Angelo E. (2007). Computational reconstruction of pacemaking and intrinsic electroresponsiveness in cerebellar Golgi cells. Front. Cell. Neurosci. 1:2. 10.3389/neuro.03.002.2007 18946520 PMC2525930

[B61] Solinas S. Nieus T. D'Angelo E. (2010). A realistic large-scale model of the cerebellum granular layer predicts circuit spatio-temporal filtering properties. Front. Cell. Neurosci. 4:12. 10.3389/fncel.2010.00012 20508743 PMC2876868

[B62] Spira M. E. Hai A. (2013). Multi-electrode array technologies for neuroscience and cardiology. Nat. Nanotechnol. 8, 83–94. 10.1038/nnano.2012.265 23380931

[B63] Tomsett R. J. Ainsworth M. Thiele A. Sanayei M. Chen X. Gieselmann M. A.. (2015). Virtual Electrode Recording Tool for EXtracellular potentials (VERTEX): comparing multi-electrode recordings from simulated and biological mammalian cortical tissue. Brain Struct. Funct. 220, 2333–2353. 10.1007/s00429-014-0793-x 24863422 PMC4481302

[B64] Trayanova N. A. Henriquez C. S. Plonsey R. (1990). Limitations of approximate solutions for computing the extracellular potential of single fibers and bundle equivalents. IEEE Trans. Biomed. Eng. 37, 22–35. 10.1109/10.43608 2154399

[B65] Vierling-Claassen D. Cardin J. A. Moore C. I. Jones S. R. (2010). Computational modeling of distinct neocortical oscillations driven by cell-type selective optogenetic drive: separable resonant circuits controlled by low-threshold spiking and fast-spiking interneurons. Front. Hum. Neurosci. 4:198. 10.3389/fnhum.2010.00198 21152338 PMC2996257

