# Supplementary material for: Computational Modeling of Single Neuron Extracellular Electric Potentials and Network Local Field Potentials using LFPsim
Source: Front Comput Neurosci. 2016 Jun 28;10:65. doi: 10.3389/fncom.2016.00065 (PMC4923190; doi:10.3389/fncom.2016.00065)
Supplement: Supplementary file 1 [file DataSheet1.PDF]

Supplementary Material

Computational Modeling of Single Neuron Extracellular Electric Potentials and Network Local Field Potentials using LFPsim

Harilal Parasuram[1], Bipin Nair[1], Egidio D'Angelo[2, 3], Michael Hines[4], Giovanni Naldi[5] and Shyam Diwakar[1*]

[1]Amrita School of Biotechnology, Amrita Vishwa Vidyapeetham (Amrita University), Amritapuri, Kerala, India.

[2]Dept. of Brain and Behavioral Sciences, University of Pavia, Pavia, Italy.

[3]Brain Connectivity Center, C. Mondino National Neurological Institute, Pavia, Italy.

[4]Dept. of Neuroscience, Yale School of Medicine, New Haven, CT, USA.

[5]Dept. of Mathematics, University of Milan, Milan (MI), Italy.

*** Correspondence:** Shyam Diwakar, Amrita School of Biotechnology, Amrita Vishwa Vidyapeetham (Amrita University), Amritapuri, Kerala, India, 690 525. Email: shyam@amrita.edu

1 LFPsim

LFPsim is a set of scripts for NEURON simulation environment to reconstruct LFP from biophysical models of neurons and networks. LFPsim uses NEURON's extracellular mechanism to calculate total ionic currents from neuronal compartments at each time step (dt). The extracellular potential at a given point (x,y,z) was calculated by setting pointers to "lfp.mod" and for multiple recording points "mea.mod". Point Source Approximation (PSA), Line Source Approximation (LSA) and RC filter techniques were implemented in LFPsim to simulate extracellular potential of a single cell and LFP of network models.

1.1 Getting familiar with LFPsim Interface

LFP simulation tool GUI was created using NEURON's graphical components. GUI mainly consisted of Morphology view window (A), Voltage changes view window (B), Reconstructed LFP view panel (C), Electrode parameters (D), extracellular medium properties set panel (D).

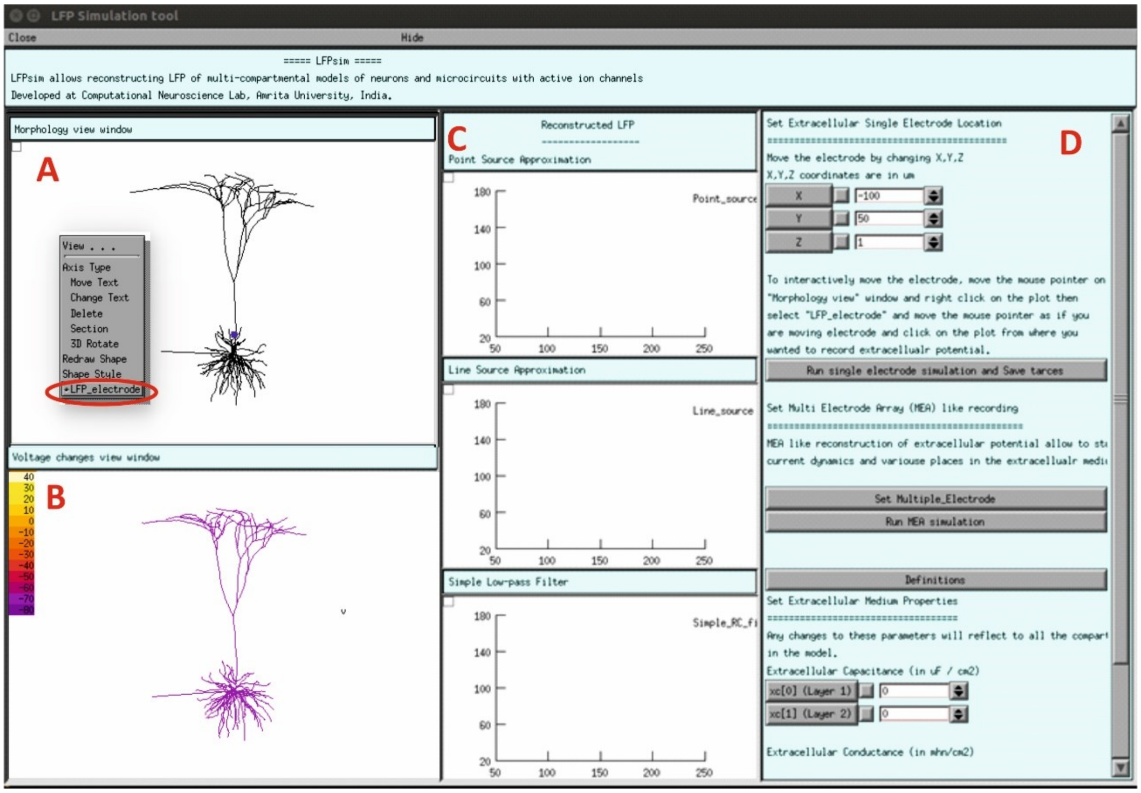

Supplementary Figure 1. LFPsim GUI. A. ***Morphology view window*** is NEURON's shape plot allowing the visualization of neuron morphology and extracellular electrode location (shown in blue). Right click of mouse on this window allows users to interactively select LFP electrode location by selecting "LFP_electrode" menu (red circle). Right click of mouse on this window will also allow options including viewing the model at different angles. B. ***Voltage changes view window*** is a NEURON's space plot to visualize voltage changes across the neuronal cable during the activity. Voltage range from -70 mV to +40 mV was defined as a colormap. C. ***Reconstructed LFP view panel***, allow users to view reconstructed LFP in three different schema PSA, LSA and RC methods. D. ***Simulation controls***, allows setting electrode parameters, extracellular medium properties and running simulation.

1.2 Computational modeling of LFP of detailed neuron models

To execute LFPsim, stepwise procedure are listed below. Please visit NEURON's website for installing the simulation environment.

Follow steps from 1 to 14 to run single electrode LFP simulation.

1. Download the LFPsim source code and biophysical model of neuron/network from ModelDB. LFPsim can also be downloaded from: https://github.com/compneuro/LFPsim. In this example, mathematical models of pyramidal neuron (deep, superficial, aspiny, stellate) by (Mainen and Sejnowski 1996) was used (ModelDB Accession ID: 2488).
2. Unzip or extract LFPsim files and model neuron/network.
3. Copy and paste all files and folder in the LFP tool folder to neuron/network model folder. Do note "lfp.mod" and "mea.mod" must be copied and placed along with model's mechanism (mod) files.

4. It is recommended to follow the model's "README" file for any specific procedures regarding compiling the model in NEURON.
5. For Linux/Unix users, opening a terminal and changing the present working directory to the neuron model folder, executing *nrnivmodl* will compile the model. If code was pre-compiled before copying "lfp.mod" and "mea.mod" files to the model directory, it is recommended to recompile after including the mod files along with other channels and receptor mod files.
6. For Windows users, double clicking on "mknrndll" and setting the path to location of the ".mod" files and clicking on "nrnmech.dll" button, compiles the mechanism files (including the mea.mod and lfp.mod files in the same directory).
7. It is recommended to follow the model's "README" file to load the neuron model in NEURON. In this example, mosinit.hoc file was used to load neuron models. On the Linux terminal, type "nrngui mosinit.hoc" to load the model in Linux. For Windows users, double clicking on mosinit.hoc file in the model directory, loads the model. Two NEURON windows popping up indicates the model was loaded. Follow the model's README, and click on "Figure 1 demo" button to load the neuron model.

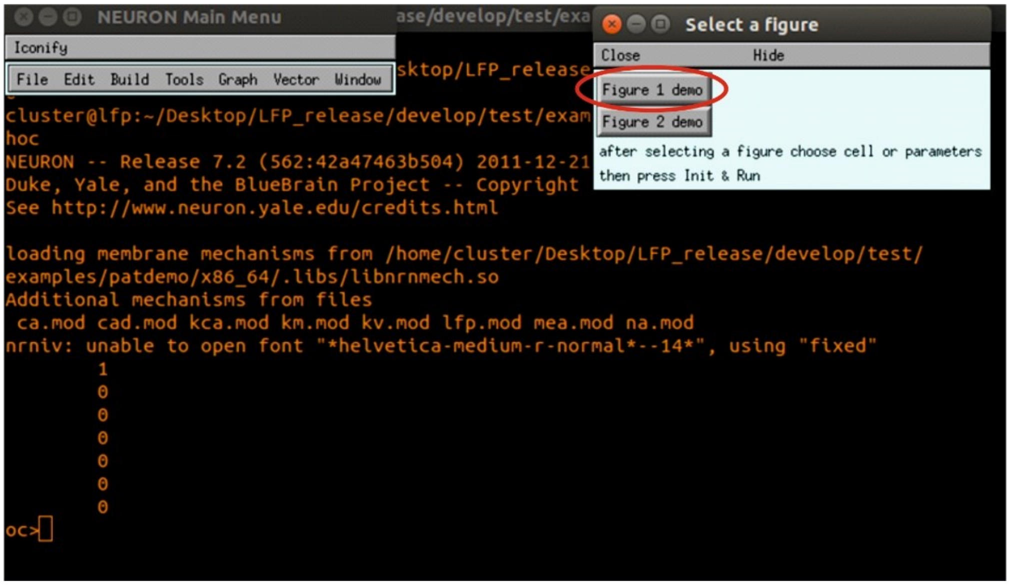

Supplementary Figure 2. Executing pyramidal neuron model.

Click on "L5 Pyramid" button to load the neuron model.

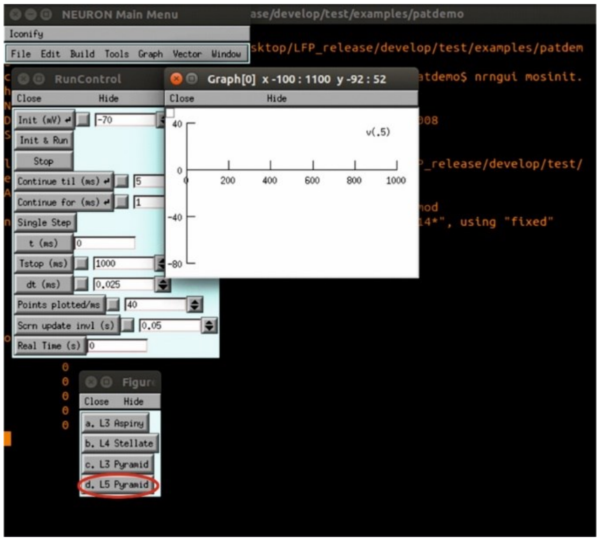

Supplementary Figure 3. Executing L5 pyramidal neuron model.

Once loaded, a shape plot will be generated as shown in Supplementary Figure 4.

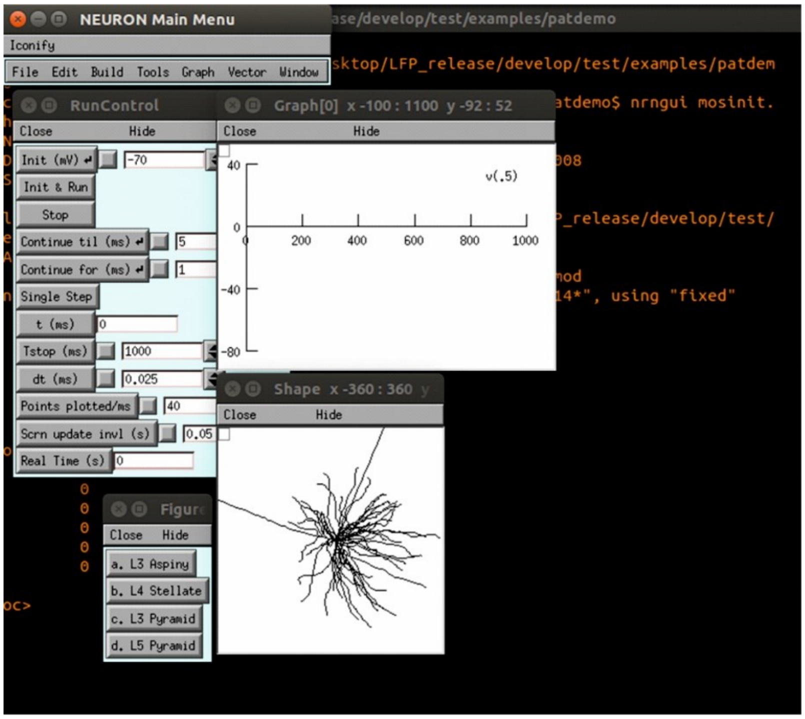

Supplementary Figure 4. L5 pyramidal neuron space plot.

After loading the model into NEURON, call "extracellular_electrode.hoc" from NEURON's terminal by typing *xopen("extracellular_electrode.hoc")* to load LFPsim in NEURON. For python based models,

from neuron import h

h.xopen("extracellular_electrode.hoc")

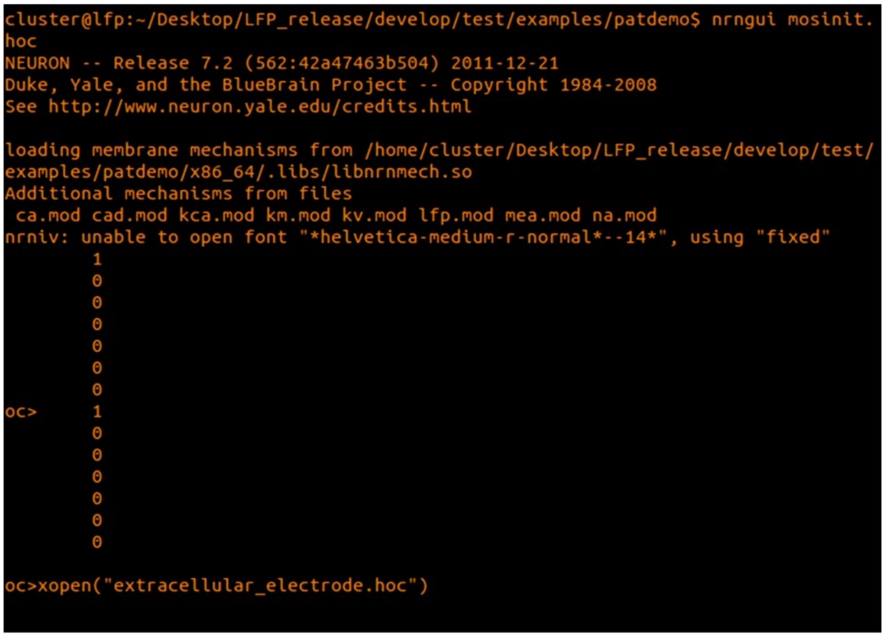

Supplementary Figure 5. Loading LFPsim with the NEURON model.

8. This invokes the LFPsim GUI on successful execution.

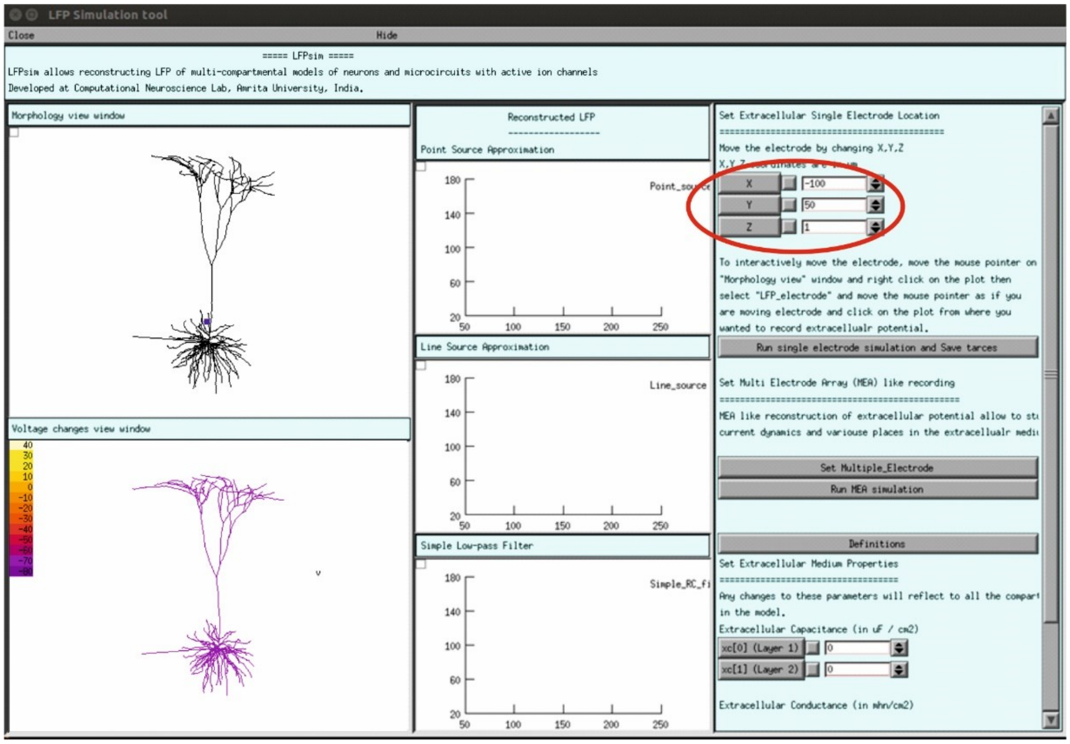

Supplementary Figure 6. LFPsim and pyramidal neuron model.

9. Set input stimuli to neuron model.
10. Users can set single electrode location both manually and interactively (see Figure 1D). To set manually, the user needs to provide values for x,y,z of electrode location in panel D (see Figure 1). To interactively change the electrode position, the mouse pointer on "Morphology view" window

may be moved and by right clicking on the plot, then select "LFP_electrode". Moving electrode is performed by moving the mouse pointer and clicking on the plot at the point of interest to record the extracellular potential.

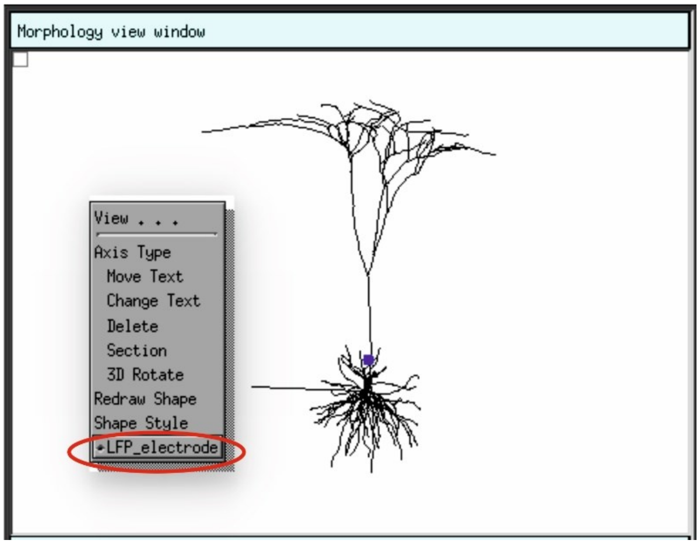

Supplementary Figure 7. Modifying LFP electrode settings.

11. Execute single LFP electrode simulation by clicking on “Run single electrode simulation and save trace” button.

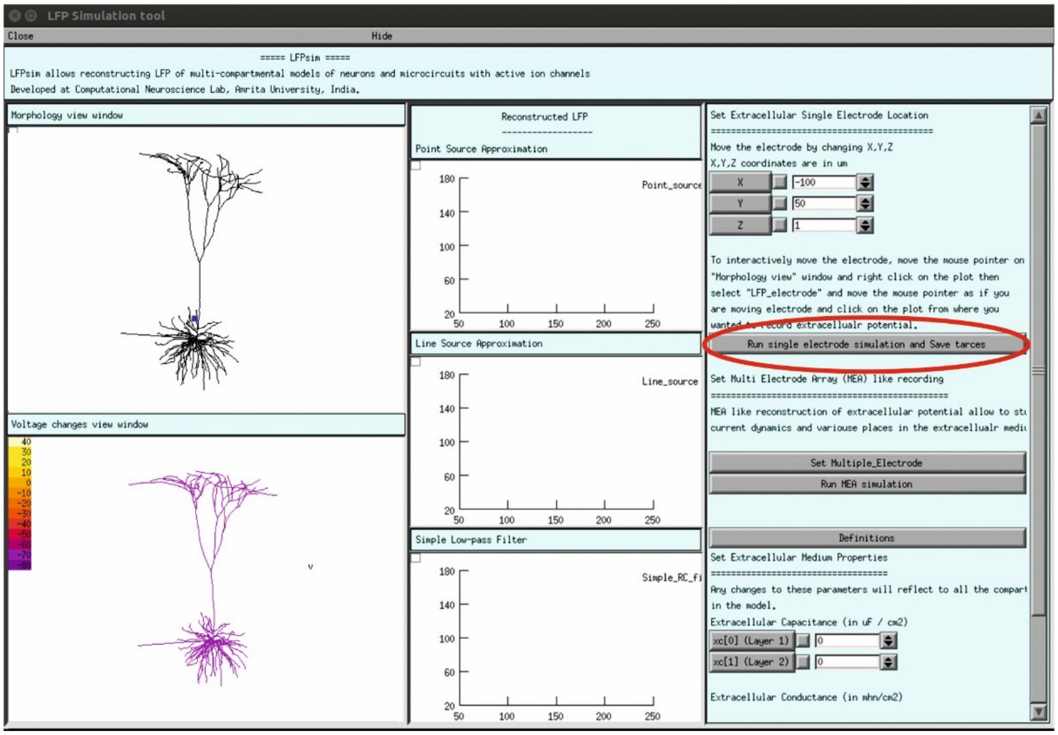

Supplementary Figure 8. Executing LFP simulation on L5 neuron model.

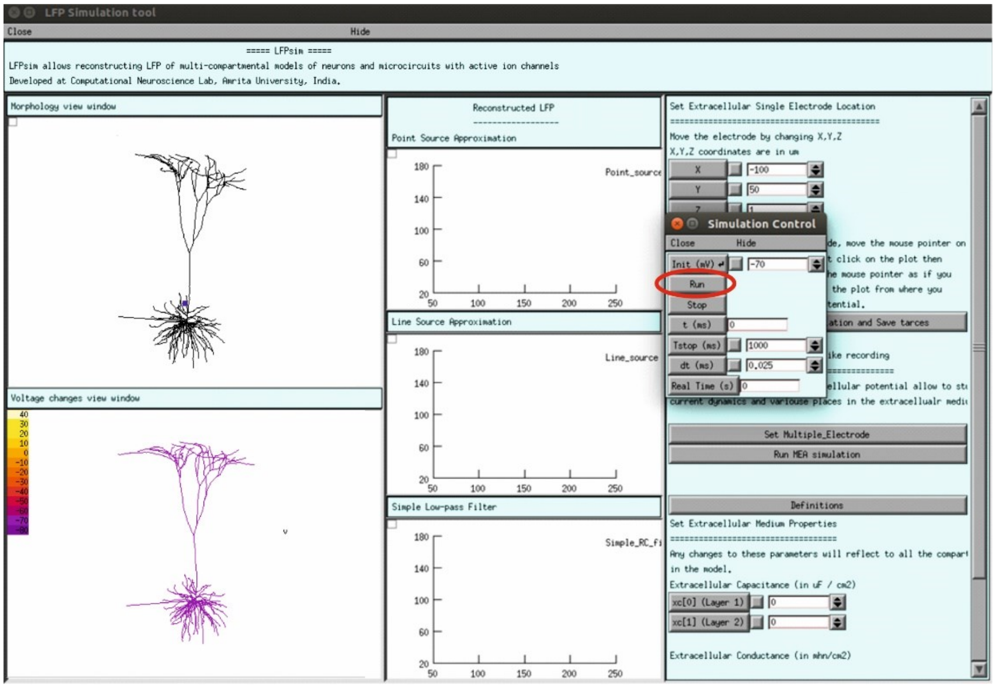

Supplementary Figure 9. Running LFP simulations.

12. When run is completed, a pop up will indicate "Simulations complete" and traces will be saved to "LFP_traces" directory within the model's directory.

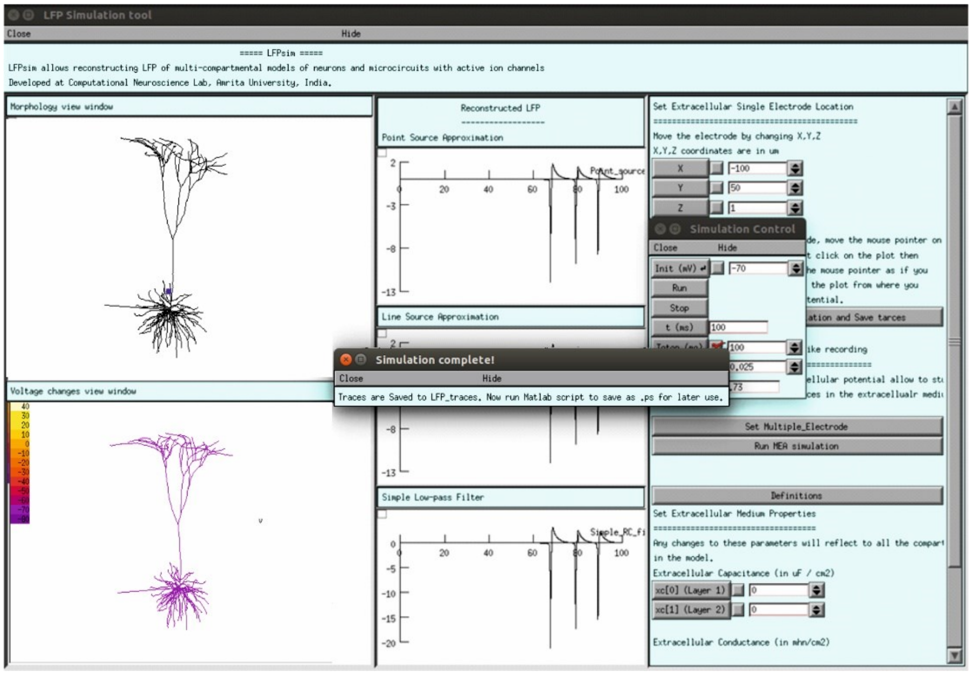

Supplementary Figure 10. Simulation complete indication in LFPsim.

13. Traces may be plot using GNU Octave, MATLAB or Python; sample scripts are provided in "LFP_traces" directory.

14. **For multiple electrode "MEA" simulation, please follow until step 10,** and Click on "Set Multiple_Electrode" button in simulation control window (see Figure 1D).

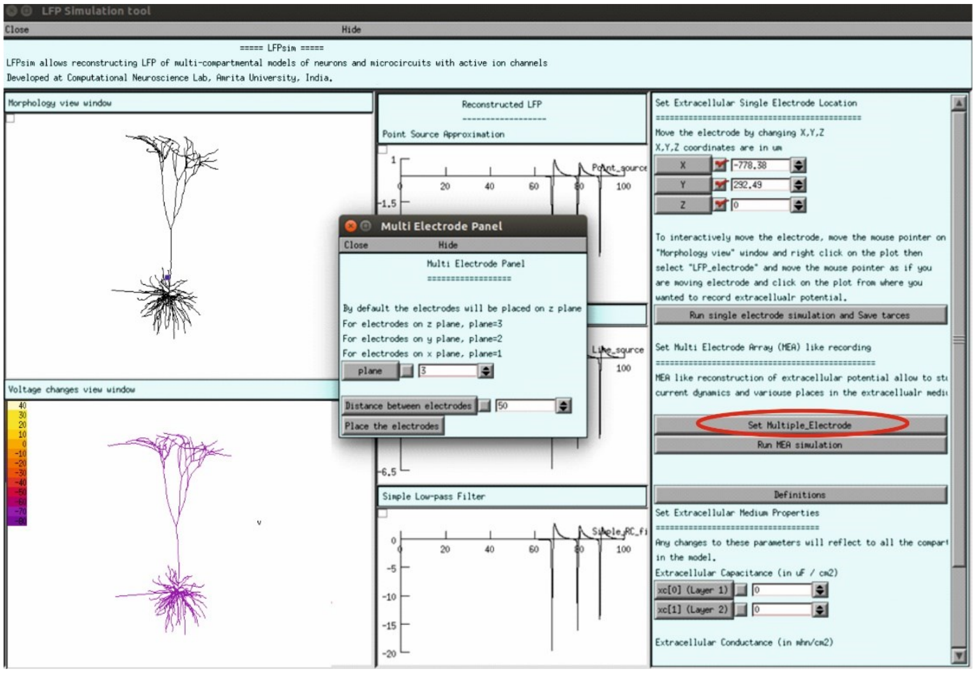

Supplementary Figure 11. MEA simulations in LFPsim.

Users can change distance between the virtual electrodes (as an example, 200µm was set). To "set" the electrode location, click on "Place the electrode" button. Users can see multiple virtual electrodes as red dots in Morphology view window.

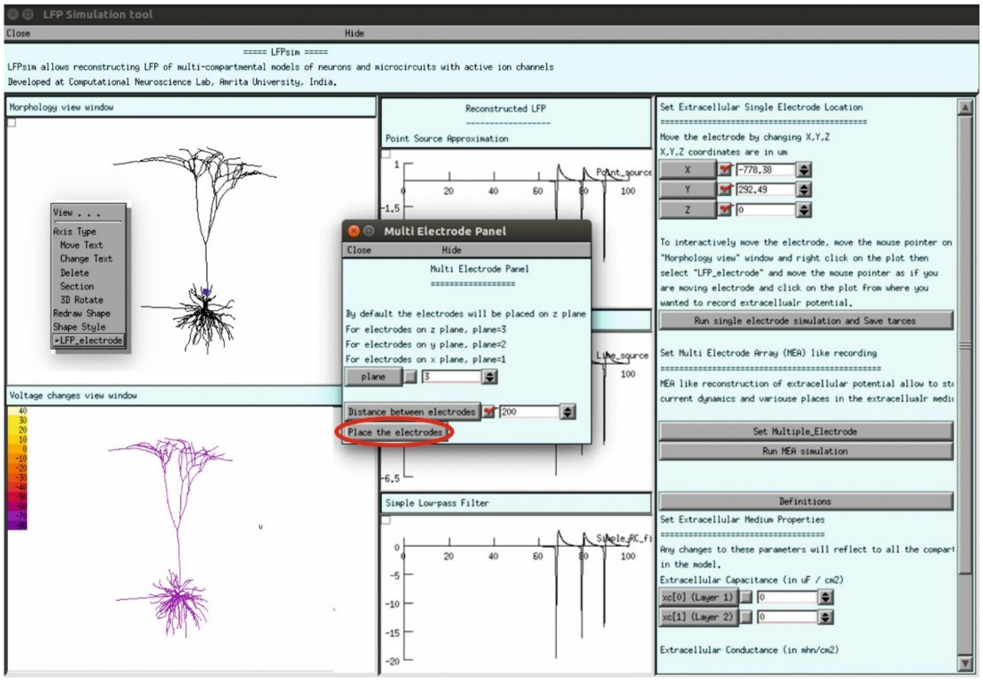

Supplementary Figure 12. “Placing” the MEA electrode.

Electrodes can also be relocated on to a different plane by changing plane variable in the panel.

15. After placing the MEA electrodes, user can execute “MEA” simulation by clicking on “Run MEA simulation” button.

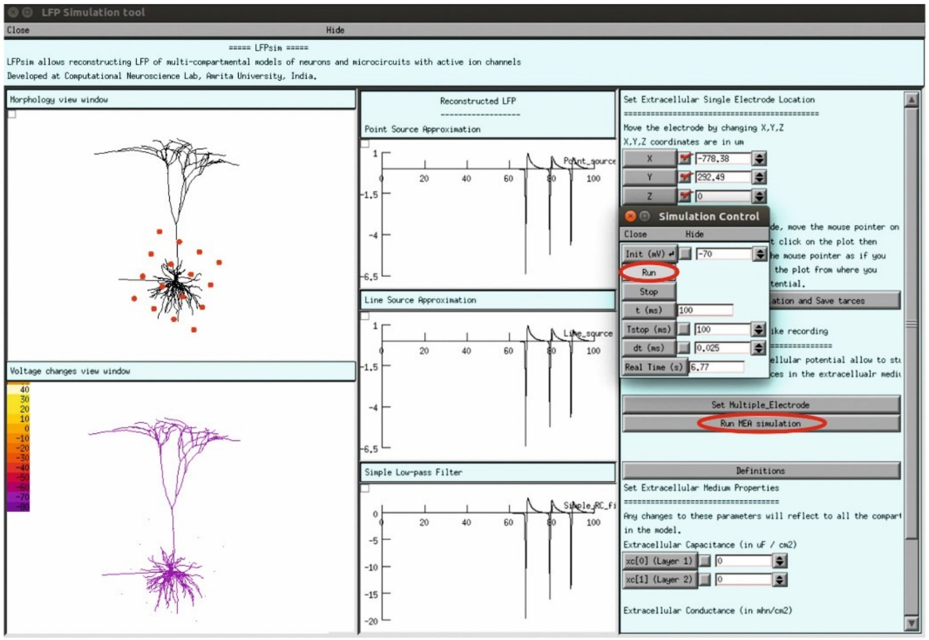

Supplementary Figure 13. Simulating “MEA”.

16. A pop up “Simulation complete” will indicate run completed and MEA traces will be saved to “LFP_traces”.

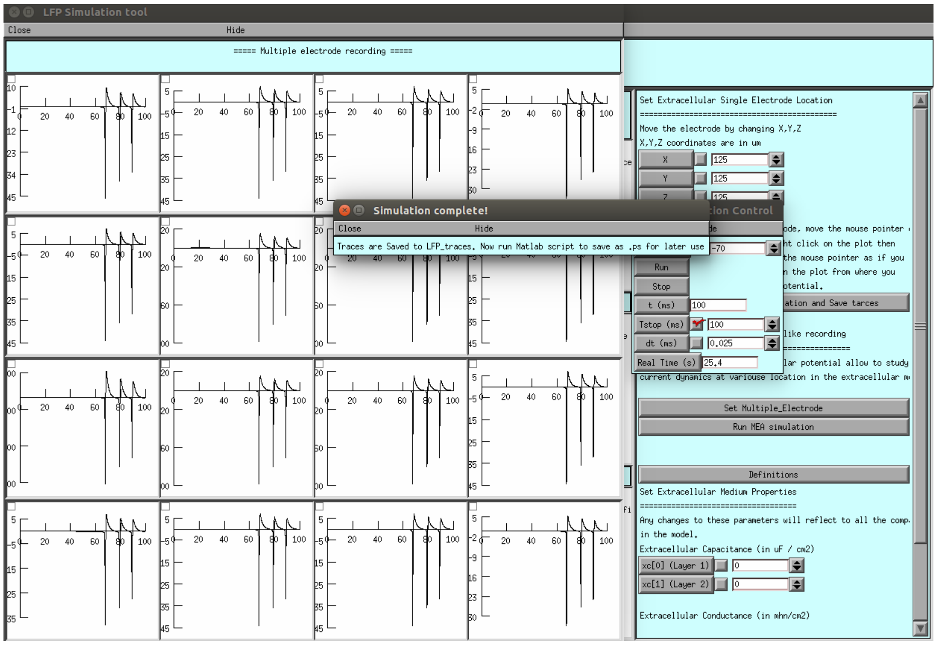

Supplementary Figure 14. “MEA” GUI in LFPsim.
